# “Surra,” or Progressive Pernicious Anæmia

**Published:** 1887-07

**Authors:** Richard W. Burke

**Affiliations:** Jubbulpore, India


### Art. XXI. "SURRA," OR PROGRESSIVE PERNICIOUS ANÆMIA.

BY RICHARD W. BURKE, A. V. D.

*Jubbulpore, India.*

Among the most important diseases in medicine are the so-called constitutional blood-disorders: Anæmia, Chlorosis, Leukæmia, and Pseudo-leukæmia—which are so little understood, also Pernicious-Anæmia, the etiology of which is still obscure. Since its discovery by Addison in 1835, and the early observations of Biermers in 1868—1872, a great deal of information regarding its general features has been made out, but chiefly through the observations of Quinke, Eichhorst, Zenker, Immerman, Ponfick and others. We may explain, very briefly, our present knowledge with respect to its etiology, symptomatology and *post mortem* appearances considered generally and specially. We recognize, in the horse as well as in the human subject, two forms of pernicious anæmia; idiopathic or essential, and symtomatic.

The causes of the first form, which is the essential disease, are altogether unknown. In general, bad feeding and stabling, and non-hygienic conditions, are the usually received causes; and, owing to this, we consider it an 'infectious disease'; or, it may be regarded as a constitutional disease of the blood, and of the red bone-marrow. All the cases which have come under observation have hitherto proved incurable.

The causes of the second form are better known. This form is caused by a certain blood-sucking intestinal worm, principally the *Ankylostomum* (*Dochmius*) *duodenale* and *Botriocephalus latus.* This was first pointed out by Perroncito, in 1880, to be the cause of progressive pernicious anæmia among workmen and others. The disease was described for the first time in India, under the name of

"Surra" as occurring commonly in horses, mules and camels, by Inspecting Veterinary Surgeon G. Evans, M. D., of the Army Veterinary department, who noted the presence of a parasite in all the diseased animals examined, and in others subjected to experimental inoculations. The nature of the parasite, and its exact pathological significance are points which Dr. Evans did not satisfactorily clear up; although, the suggestion subsequently made by Veterinary Surgeon Steel, that it was a spirillum, would not be accepted by him, who maintained that whatever it might be, it was not a member of the family of bacteria. Dr. Edgar M. Crookshank, who carefully studied the parasite in 'surra' blood, agrees with Dr. Evans in considering it to be *not* a sprillum, and proposes to name it after its discoverer, *Trichomonas evansi.* But the relation of this parasite to the disease has remained an unsettled question.

The remarkable tendency of the blood to harbor parasites has been a matter of not recent observation. Their characteristic features manifest a disposition to augment in numbers under any shock or depression of the system. Sometimes, prolonged exposure to malarious and ill-sanitary influence, debilitating effects from impaired nutrition, from hyper-pyrexia, etc., issues in a morbid alteration of the blood peculiarly fitted for the growth and multiplication of these organisms; but, even when no change in the surroundings of the animal is discoverable, a deep impression is often stamped upon the system, leading to their increase. In a recent inquiry it has been shown that these organisms are to be found in no less than 25 per cent. of apparently healthy animals.

These parasites are usually classed as filaria, and they do not seem to excite any disease when in moderate numbers. In other cases, apoplexy may result from embolism of the heart and closure of the valves; and also the embryos appear to cause apoplexy by blocking up the capillaries of the brain. Besides this they may cause epileptiform convulsions and anæmia and wasting.

In all kinds of animals, then, this parasite must exist, ready to resume its active form whenever the conditions of climate, of weakness and ill-health presents themselves afresh. Without doubt, the parasites which are the cause of this disease are widely prevalent, and exist in the blood of most animals, but attenuated; and in this state an animal may harbor them in its blood, without showing much, if any illness. They only become dangerous when, through overcrowding, and other causes, in bodies enfeebled by disease their virulence becomes exaggerated. Under weakened states of the body, as in 'surra,' doubtless the parasites multiply rapidly, and even assume pathogenic properties, under favorable conditions. It should also be stated that the parasite in 'surra' has never been isolated apart from the blood, and the disease then produced by inoculating healthy animals with it. It is most probable therefore that the parasites in 'surra' are only associated with the disease, the impoverished blood affording a suitable nidus for their development.

*Pathology.*—Zschokke first decribed this disease under the name of pernicious anæmia of the horse, and also noted the presence of spiral organisms in the blood, the same as those observed by Klebs and Frankenhauser in the human subject. The presence of these organisms, he considers, accounts for the infectiousness of the disease. Fröhner also saw organisms in the blood of the horse between the red corpuscles: they were from $\frac{1}{3}$ to $\frac{1}{2}$ the size of a red blood corpuscle, were not quite so thick, but usually grouped together in fours and sixes.

M. Netter shows that these micro-organisms found in the alimentary canal in its normal state are also found in the bile duct; they penetrate the small ducts of the liver, and pass from thence into the blood (*Archives de Medecine*, 1884). Veterinary Surgeons of the Army, on the continent of Europe, have generally described it under the head of lung disorders of an infectious type. Until my own observations were published (*Supplement to Report on Remittent Anthrax, 5th March*, 1887), veterinarians in India have treated it under different names, as 'surra,' relapsing fever, etc., according as it suited the fancy of different writers.

Other animals, besides the horse, have been known to suffer from the disease. Megnin describes a symptomatic type of this disease seen in dogs and cats, which was caused by an ankylostome producing anæmia, and which is, without doubt, a similar disease to the symptomatic form of anæmia in man. Johne saw this disease in the dog as a secondary affection following a supperative form of disease. Imminger has observed enzootic outbreaks of it in cattle. Fröhner describes having only recently seen cases of this disease in the horse, and Friedburger also saw an outbreak of it in the same animal. The cases described by Dieckerhoff, under the name of "scalma," are undoubtedly the same disease. The latter author mentions nine cases which, although of a milder nature, showed symptoms allied to those of pernicious anæmia. Dieckerhoff considered it infectious.

*Etiology.*—Dr. Ponfick's experiments show that pernicious anæmia may be experimentally produced in dogs and rabbits by administering blood-dissolving agents to them for some weeks, but especially glycerine, pyrogallic acid, etc., which always produced a state of lethargy and extreme weakness in these animals, the mucus membranes becoming pale and anæmic, the action of the heart very irregular, with blowing sounds heard on auscultation of the latter organ, the pulse also weak, and the temperature raised. The excrements were sometimes mixed with blood. A microscopic examination of the blood showed a nearly normal condition of things, in the first week; from the third to the fourth week, the blood became pale and watery, with an excess of white cells in it. On dissection of the body, marked anæmia was found, with fatty degeneration of the muscles of the heart, of the intima of the veins, the liver, and kidneys; and blood-vascular extravasations in internal organs, but principally in serous membranes, in the lungs, the brain, the spinal marrow, subcutis, etc.

Professor Ponfick thinks that the blood-dissolving agents cause separation of the hœmoglobin from the red blood-corpuscles, leading to marked disturbances in the relative proportion of the blood constituents. The leucocytosis, which is a marked feature of pernicious anæmia, is also characteristic of hæmoglobinuria, and which in acute and subacute cases is always present. An excess of free hœmoglobin in the spleen, liver, and kidneys may probably be a cause of swelling of these organs seen in this disease. The continued loss of hæmoglobin from the blood interferes with the formation of new blood in a very serious manner. The author has proved by further examinations that free hæmoglobin in the blood destroys the white cells and promotes the growth of fibrin ferments. Large quantities of these ferments cause a marked disturbance of the circulation, the blood flows slower than under normal conditions, which necessarily interferes with the proper function of the blood-forming organs. The body becomes poorer and poorer through the large numbers of the red blood-corpuscles becoming destroyed in this disease, and through loss of hæmoglobin and oxygen; which cause defective nutrition leading to excessive deposition of fat in the principal organs of the body, and which is the cause again of hæmorrhages in different organs.

One cannot refrain from pointing out the very meagre evidence we possess, in the face of these experiments, with regard to the share taken by the parasites met with in the blood of surra. The results of these experiments at once suggest to our mind an explanation with regard to the parasites as a consequence of impoverished blood in this disease.

Dr. Crookshank states that the closest examination has confirmed this belief "that the parasites found in the blood of healthy rats are morphologically identical with the stained parasites of surra." Dr. Evans noted the presence of filaria of different species in the blood of diseased as well as healthy camels (*Veterinary Journal*, *July*, 1881), and Crookshank notes the same fact in the case of filaria found in the blood of rats. Since all observations prove the existence of filaria of different species in the blood to be compatible with health, we have no reason for wonder when we find them greatly increased during disease.

It should be further stated, as Dr. Crookshank points out, that the organism has never been isolated apart from the blood in surra, and the disease then produced by inoculation of healthy animals,—a point of considerable moment in the etiology of this disease.

*Symptoms.*—The appearance during life are principally those of a general loss of blood, indicated by anæmia of mucous membranes, languor, dyspnœa, a weak and readily excitable pulse, palpitatation of the heart, and fever. There is increasing debility, with little or no loss of appetite. In spite of a greedy appetite, debility appears progressive in character. Dropsical swellings usually occur towards the latter stage of the disease. The malady is usually progressive, seldom acute in character. The course is prolonged to several, usually six to eight weeks.

With regard to the fever accompanying this disease, Mr. Steel has noted an unlimited number of remissions on the 4th and 5th days usually, which he mistook for the remissions seen in relapsing fever. Zschokke has shown that the fever accompanying these cases is of an intermittent (*quartan*) type. Fröhner has likewise noted a marked rise in temperature on the 4th day, which, with slight remissions, remained high till death. He also saw partial paralysis, or an increasing weakness of the hind quarters in horses the subject of this disease, a symptom which was often observed in the Burmah outbreak. Zschokke mentions jaundice as another frequent symptom in the horse, as well as enlargement of the lymphatic glands and punctiform extravasations—symptoms commonly noted in surra in India. The urine is albuminous in character, and of an acid re-action.

*Post Mortem Appearances.*—On dissection, a general anæmia, together with fatty metamorphosis, unattended by other changes, is noted. Frequently, though not always, hæmorrhages take place more or less in all the organs, but principally in the serous membranes, the muscles, the retina, and the larger glands. The spleen and liver are sometimes swollen, and contain thrombi in the larger vessels. The marrow in the bones appears altered in character, and presents a jelly like aspect, containing innumerable granular blood-corpuscles. The liver and other organs contain an excess of iron-salt. The true pathological cause is to be found in the blood; the red blood-corpuscles are decreased in number and altered in shape, size and aspect frequently presenting a markedly serrated outline. Nodular blood particles are also to be seen in the blood. In short, the blood is in a state of complete disorganization.

Zschokke states that none of the principal organs show any marked structural change. Besides anæmia, he has noted hæmorrhages under the serous membranes, swelling of the liver and spleen, loss of striæ in the muscular fibres of the heart, blood extravasations in the marrow of the bones, and a decrease in the number of red corpuscles in the blood, the same as in pernicious anæmia in man. He also noted the presence of spiral organisms in the blood.

Mr. Oliphant, Principal Veterinary Surgeon in India, in his G. O., dated 12th Febuary, 1887, writes :—"In one outbreak of "surra" in the 18th Bengal Cavalry, in which 180 horses died, I made dozens of *post mortem* examinations, and the appearances in all were identical—extreme pallidity of all the tissues, with perhaps a trifling serious effusion into the abdomen, etc. In fact, the animals looked as if they had been starved to death." Dr. Evans was also most careful in pointing out that absence of any marked structural changes was peculiar to surra. The other symptoms, such as the adipose degeneration of the liver, spleen, heart and other organs, the general hæmorrhagic diathesis, etc., may be regarded as secondary in origin.

*Diagnosis*.—The marked anæmic conditions observed, both *ante* and *post mortem*, the leucocytosis, fatty degeneration of the muscles, liver, spleen, kidneys and other organs, the general hæmorrhages, and the chronic state of fever,—which end in death in so many cases, leave no doubt as to the nature of the disease.

*Treatment*.—In all forms of serous anæmia, authors have always attached the greatest importance to treatment by arsenic; and in a recent paper by Professor Osler, in the *Therapeutic Gazette*, he states that in all cases of pernicious or essential anæmia there was no one case of recovery in which arsenic did not form the basis of treatment. It is not, however, a specific. Iron is only occasionally useful in these cases. Mr. Steele also mentions these remedies in the treatment of "surra" although he met with no greater success than usually follows their use in pernicious anæmia in man. Exercise would appear to be more favorable to the recovery in these cases than absolute rest. Change of climate has been found occasionally useful, both in the case of man and in that of the lower animals. Sir Joseph Fayer—who even suggests that "beri-beri" in man is probably one form of pernicious anæmia—thinks that a hilly climate is particularly suitable for the less advanced cases, although he admits its efficacy in more prolonged cases to be somewhat doubtful, if not altogether *nil.*

The whole subject of parasites and parasitism in equine medicine, is one which has yet to be studied completely; but, as a clinical observer, I believe that I can recognize in "surra" a pathological state which has a curious relation to pernicious anæmia in man, which I have endeavoured to bring out in this paper for the benefit of English-speaking veterinarians in India in particular, and to associate it with that disease of the lower animals long since recognized by veterinarians on the continent of Europe, but especially by Megnin, Johne, Imminger, Zschokke, Friedberger, Fröhner and others.

